# Arabidopsis K^+^ transporter HAK5-mediated high-affinity root K^+^ uptake is regulated by protein kinases CIPK1 and CIPK9

**DOI:** 10.1093/jxb/eraa212

**Published:** 2020-06-02

**Authors:** Alberto Lara, Reyes Ródenas, Zaida Andrés, Vicente Martínez, Francisco J Quintero, Manuel Nieves-Cordones, M Angeles Botella, Francisco Rubio

**Affiliations:** 1 Departamento de Nutrición Vegetal, Centro de Edafología y Biología Aplicada del Segura-CSIC, Campus de Espinardo, Murcia, Spain; 2 Department of Plant Developmental Biology, Centre for Organismal Studies, Heidelberg University, Heidelberg, Germany; 3 Instituto de Bioquímica Vegetal y Fotosíntesis, CSIC-Universidad de Sevilla, Américo Vespucio, Sevilla, Spain; 4 Departamento de Biología Aplicada, Universidad Miguel Hernández, Alicante, Spain; 5 Bielefeld University, Germany

**Keywords:** Arabidopsis, AtCBL, AtCIPK, AtHAK5, potassium, uptake

## Abstract

The high-affinity K^+^ transporter HAK5 is the major contributor to root K^+^ uptake from dilute solutions in K^+^-starved Arabidopsis plants. Its functionality is tightly regulated and its activity is enhanced under K^+^ starvation by the transcriptional induction of the *AtHAK5* gene, and by the activation of the transporter via the AtCBL1–AtCIPK23 complex. In the present study, the 26 members of the Arabidopsis CIPK protein kinase family were screened in yeast for their capacity to activate HAK5-mediated K^+^ uptake. Among them, AtCIPK1 was the most efficient activator of AtHAK5. In addition, AtCIPK9, previously reported to participate in K^+^ homeostasis, also activated the transporter. In roots, the genes encoding AtCIPK1 and AtCIPK9 were induced by K^+^ deprivation and *atcipk1* and *atcipk9* Arabidopsis KO mutants showed a reduced AtHAK5-mediated Rb^+^ uptake. Activation of AtHAK5 by AtCIPK1 did not occur under hyperosmotic stress conditions, where AtCIPK1 function has been shown to be required to maintain plant growth. Taken together, our data contribute to the identification of the complex regulatory networks that control the high-affinity K^+^ transporter AtHAK5 and root K^+^ uptake.

## Teaser

Arabidopsis protein kinases CIPK1 and CIPK9, in addition to CIPK23, regulate K^+^ transporter HAK5-mediated K^+^ uptake, and their genes were induced by K^+^ deprivation.

## Introduction

Potassium (K^+^) is an essential macronutrient for plants. Its acquisition by the root from the soil solution is a crucial process for plant growth and development because K^+^ fulfills important functions within the plant cells (White and Karley, 2010). To accomplish these functions, the cytosolic K^+^ concentration should be maintained constant, around 100 mM (Leigh and Wyn Jones, 1984), which contrasts with the highly variable K^+^ concentrations of the soil solution. In many cases, the K^+^ concentration gradient across the root cell plasma membrane is several orders of magnitude. In spite of this, K^+^ accumulation in the cell is ensured by the operation of specific K^+^ transport systems and by the existence of the electrochemical H^+^-gradient across the plasma membrane generated by the H^+^-ATPase (White and Karley, 2010) that drives K^+^ entry into the cell.

In Arabidopsis, two transport systems, AtHAK5 and AKT1, are the major contributors to root K^+^ uptake. The Arabidopsis model can be, to some extent, applied to other plant species (Nieves-Cordones *et al.*, 2016). Depending on the external K^+^ concentration, each of these two systems dominates K^+^ uptake. This requires a fine-tuned regulation of AtHAK5 and AKT1 activities. At very low external K^+^ concentrations, below 20 µM, the high-affinity K^+^ transporter, AtHAK5, is fully active and it is the only system mediating K^+^ uptake (Rubio et al., 2008, 2010; Pyo *et al.*, 2010). The increase in AtHAK5 activity at low external K^+^ is achieved by the transcriptional activation of the *AtHAK5* gene (Ahn *et al.*, 2004; Nieves-Cordones *et al.*, 2010) and by the activation of the transporter by AtCIPK23-mediated phosphorylation (Ragel *et al.*, 2015).

Because plants are sessile organisms, regulation of the activity of the transport systems involved in the acquisition of essential nutrients is of paramount importance for plant growth, development, and adaptation under a constantly changing environment. Members of the Calcineurin B-Like Protein-Interacting Protein Kinase (CIPK) family are emerging as pivotal elements of the regulation of transporters for different nutrients in plants and in stress responses. These kinases form complexes with Ca^2+^ sensors of the Calcineurin-B Like (CBL) protein family and phosphorylate the final targets for their activation (Pandey *et al.*, 2014; Cherel and Gaillard, 2019). Some of these kinases, such as AtCIPK23, regulate several transport systems and, conversely, a given transporter can be activated by different AtCIPKs (Pandey *et al.*, 2014). Identifying the elements involved in the regulation of the transport systems will deepen our knowledge of the mechanisms involved in plant nutrition and stress tolerance, and provide tools for their improvement.

In the present study, we have screened the 26 Arabidopsis CIPKs for their capacity to activate AtHAK5. Several members of this family of kinases promote activation of AtHAK5-mediated K^+^ uptake in yeast. Among them, we identified AtCIPK1 and AtCIPK9 which, in cooperation with AtCBL1 or AtCBL9, regulate root high-affinity K^+^ uptake via AtHAK5. The results add more evidence to the idea that a complex regulatory network modulates root K^+^ acquisition via AtHAK5.

## Materials and methods

### Plant material and growth conditions

Arabidopsis WT ecotype Col-0 and its described mutants *athak5-3*, *akt1-2*, *atcipk23-5*, *athak5-3atcipk23-5*, and *akt1-2atcipk23-5* were used (Ragel *et al.*, 2015). The *atcipk1-1* (D’Angelo *et al.*, 2006) and the *atcipk9-1* (Liu *et al.*, 2013) mutants have been described previously and were kindly provided by Dr J. Kudla (University of Münster) and Dr Y. Wang (China Agricultural University), respectively. The double mutants *athak5-3atcipk1-1*, *akt1-2atcipk1-1* and *atcipk23-5atcipk1-1* were obtained here by crossing the corresponding single mutants. Plants were grown hydroponically as described previously (Nieves-Cordones *et al.*, 2019) in a modified 1/5 Hoagland solution that contained the following macronutrients (mM): 1.4 KCl, 1.4 Ca(NO_3_)_2_, 0.35 MgSO_4_, and 0.1 Ca(H_2_PO_4_)_2_, and the following micronutrients (μM): 50 CaCl_2_, 12.5 H_3_BO_3_, 2 MnSO_4_, 1 ZnSO_4_, 0.5 CuSO_4_, 0.1 H_2_MoO_4_, 0.1 NiSO_4_, and 10 Fe‐EDDHA. To starve the plants of K^+^, a nutrient solution without KCl was used. To impose a hyperosmotic stress treatment, mannitol was added to the growth solution to reach a final concentration of 300 mM. For that purpose, plants were grown under control 1/5 Hoagland (1.4 mM K^+^) solution conditions for 28 d and then starved of K^+^ for 12 d. During this 12 d time period of K^+^ starvation, mannitol concentration was increased progressively (to 150 mM at day 11 and 300 mM at day 12). Plants were grown in a growth chamber under controlled conditions with a photon flux density of 120 µmol m^−2^ s^−1^ and 8/16 h light/dark photoperiod, at 22 °C and 65% relative humidity.

### Yeast and bacterial strains, growth and plasmids

The *trk1 trk2* 9.3 yeast strain (*MATa*, *ena1∆::HIS3::ena4∆*, *leu2*, *ura3*, *trp1*, *ade2*, *trk1∆*, *trk2::pCK64*; Bañuelos *et al.*, 1995), deficient in its endogenous K^+^ uptake systems, TRK1 and TRK2, was used. The TOP10 *Escherichia coli* bacterial strain was used for routine plasmid propagation. The cDNAs of the 26 Arabidopsis CIPKs were cloned into the yeast expression vector pGPD414 (Mumberg *et al.*, 1995). *AtCBL1* and *AtCBL9* cDNAs were cloned into pYPGE15 yeast expression vector and the AtHAK5 cDNA under the control of the PMA1 promoter was cloned into the pRS425 (Ragel *et al.*, 2015) yeast vector. Plasmids were transformed into yeast as described previously (Elble, 1992). Synthetic SD media (Sherman, 1991) were used for transformant selection. The minimal AP medium (Rodríguez-Navarro and Ramos, 1984), supplemented with different amounts of K^+^ as indicated, was used for yeast complementation assays. For that purpose, yeast suspensions were brought to OD_550_ of 1 and then 10 µl drops of serial dilutions of the suspensions were inoculated on plates with solid AP medium and incubated at 28 °C.

### Real-time qPCR

Total RNA was extracted from Arabidopsis roots by using the Macherey-Nagel NuecloSpin RNA Plant kit (Neumann-Neander, Germany) and treated with DNA-free™ kit (Thermo Fisher Scientific, Waltham, MA, USA) to eliminate contaminating DNA from the RNA samples. cDNA was synthesized with the High Capacity cDNA Reverse Transcription Kit (Thermo Fisher Scientific). Real-time PCR was performed in a 7500 Real-Time PCR System (Thermo Fisher Scientific), using default cycle settings. Relative expression levels of *AtCIPK1*, *AtCIPK9*, *AtCIP23*, and *AtHAK5* are given as log_2_(fold-change (FC)), and FC was determined by the 2^−^*^∆∆^*^*C*t^ method (Livak and Schmittgen, 2001) using the *AtPP2A* gene as reference gene (Czechowski *et al.*, 2005); the WT 1.4 mM K^+^ treatment could be used as the calibrator sample as no significant differences in the *C*_t_ values among the different plant lines were observed for the reference gene. The primers used are listed in Supplementary Table S1 at *JXB* online.

### Rb^+^ uptake experiments in plants

Plants were grown in the described 1/5 modified Hoagland solution for 28 d and then for 12 d in a solution without K^+^. Then, plants were transferred to a solution with no K^+^ containing 20 µM RbCl for 6 h. The use of Rb^+^ as a tracer for determining root K^+^ uptake has been described in previous studies (Ragel *et al.*, 2015). After the 6 h of incubation in the presence of Rb^+^, plants were harvested, separated into roots and shoots and dried at 65 °C for 3 d. Then, the dry weights of roots and shoots were determined and the plant organs were subjected to digestion with HNO_3_–HClO_4_ (2:1, v/v). Rb^+^ content in roots and shoots was determined by inductively coupled plasma (ICP) mass spectrometry by using an Iris Intrepid II ICP spectrometer (Thermo Electron Corp., Franklin, MA, USA). For each repetition, the Rb^+^ accumulated in the plant was calculated by the addition of the total Rb^+^ accumulated in the root and in the shoot. Rb^+^ uptake rates were determined by the accumulation of Rb^+^ in the plants per gram of root dry weight and unit time.

### Statistical analysis

Analysis of variance was performed with Statistix V.8 for Windows (Analytical Software, Tallahassee, FL, USA). To perform the statistical analyses, gene expression data were expressed as log_2_FC (Rieu and Powers, 2009). The differences in means were compared by using Tukey’s multiple range test (*P*<0.05).

## Results

### Several CIPKs activate AtHAK5 in yeast irrespective of their phylogenetic group

**Fig. 1.**
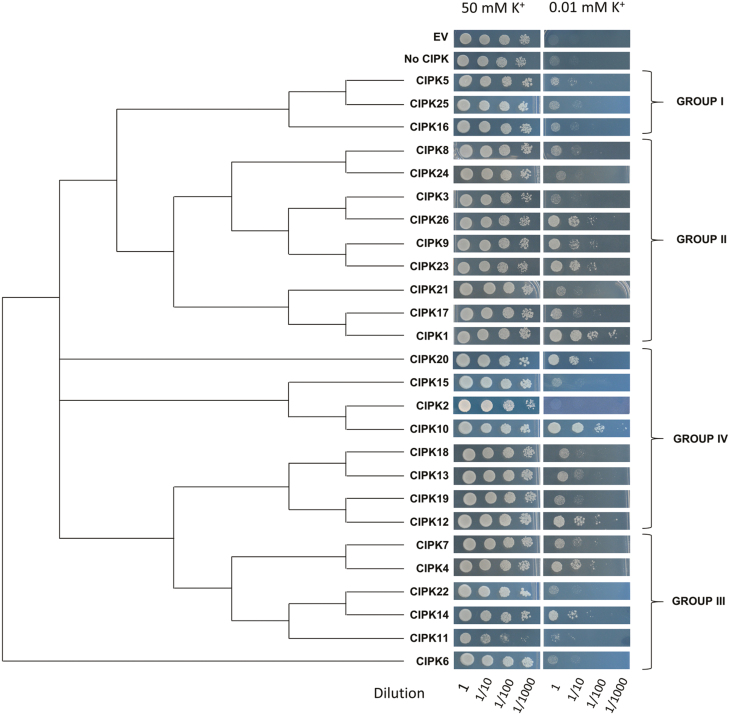
Growth tests for yeast expressing AtHAK5, AtCBL1, and the 26 Arabidopsis CIPKs. Yeast strain 9.3, deficient in the endogenous K^+^ uptake systems, was transformed with vectors containing AtHAK5, AtCBL1, and the 26 AtCIPKs. Control strains were transformed with the empty vectors (EV) or with AtHAK5 and AtCBL1 (No CIPK). Ten microliter drops of decimal dilutions of yeast transformant suspensions were grown on minimal AP medium supplemented with 50 or with 0.01 mM K^+^. Images of yeast transformed with the different kinases are arranged according the AtCIPK family phylogenetic tree. The four phylogenetic groups of the family are indicated.

To identify CIPKs other than AtCIPK23 that activate AtHAK5, the 26 Arabidopsis CIPK protein kinases were co-expressed together with the Ca^2+^ sensor AtCBL1 and the K^+^ transporter AtHAK5 in a yeast *trk1 trk2* mutant impaired in K^+^ acquisition. AtCBL1 was chosen for the initial screening because, together with AtCBL9, it was previously reported to contribute to low-K^+^ tolerance in Arabidopsis plants (Xu *et al.*, 2006; Cheong *et al.*, 2007) and to activate AtHAK5 in yeast in cooperation with AtCIPK23 (Ragel *et al.*, 2015). Drop test assays with yeast transformants under K^+^-sufficient (50 mM) and K^+^-limiting conditions (0.01 mM) were used to evaluate yeast growth and the complementation capacity promoted by the different kinases. To determine possible relationships between the phylogeny of the Arabidopsis CIPK family and the complementation capacity of each CIPK, a phylogenetic tree of the 26 Arabidopsis CIPKs was obtained. The results of the drop test for each kinase were displayed according to the obtained CIPK phylogenetic tree (Fig. 1). Control transformants included yeast transformed with the empty vectors and yeast transformed with AtHAK5 and AtCBL1. The results showed that all yeast strains grew well in media with a high concentration of K^+^ (50 mM). At the low K^+^ concentration (0.01 mM K^+^), the yeast mutant expressing the empty vectors (EV) and that transformed with AtHAK5 together with AtCBL1 (labeled in the figure as No CIPK) did not grow. By contrast, expression of the AtHAK5 transporter together with AtCBL1 and AtCIPK23 promoted a vigorous yeast growth. These results are in agreement with previous studies (Ragel *et al.*, 2015) and showed the activation of the AtHAK5 K^+^ transporter by the AtCBL1–AtCIKP23 complex in yeast. When growth of the yeast strains expressing AtHAK5 together with AtCBL1 and the different AtCIPKs was analysed, it could be observed that the 26 AtCIPKs produced different levels of complementation. Some of them, such as AtCIPK2, 3, 6, 11, 15, 21, 22, 24, and 25, did not improve growth. Others, such as AtCIPK5, 8, 13, 16, 17, 18, and 19, produced a slight improvement of yeast growth. Some CIPKs, such as AtCIPK4, 7, 9, 10, 12, 14, 20, and 26, improved yeast growth and AtCIPK1 was the one that improved yeast growth the most. Because AtCIPK9 was previously shown to play an important role in K^+^ homeostasis (Pandey *et al.*, 2007; Liu *et al.*, 2013; Singh *et al.*, 2018; Yadav *et al.*, 2018) and AtCIPK1 promoted the most vigorous yeast growth (Fig. 1), these two kinases were chosen for further characterization.

**Fig. 2.**
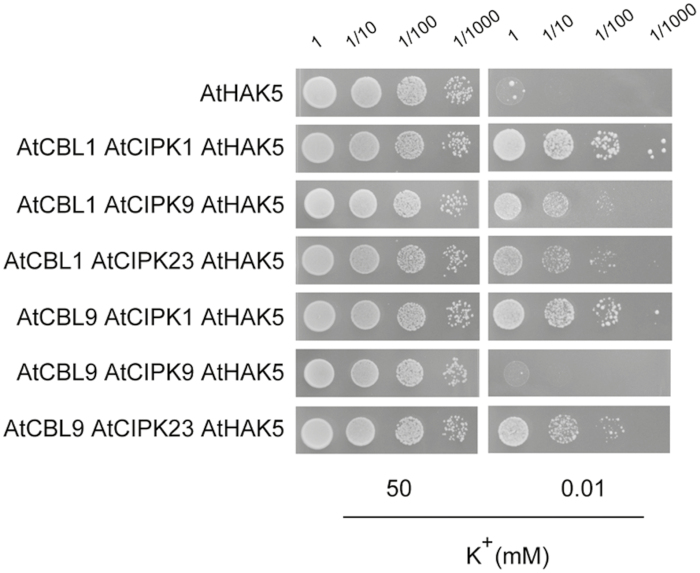
Growth tests for yeast expressing AtHAK5 and the indicated combinations of AtCIPK1, AtCIPK9, AtCIPK23, AtCBL1, and AtCBL9. The growth assay was performed as indicated in Fig. 1 with yeast transformed with expression vectors for the indicated proteins.

The described complementation assays (Fig. 1) were performed with the AtHAK5 transporter and the AtCBL1 Ca^2+^ sensor. However, in addition to AtCBL1, AtCBL9 has also been described to interact with AtCIPK1 (D’Angelo *et al.*, 2006) as well as with AtCIPK9 (Liu *et al.*, 2013; Yadav *et al.*, 2018). Therefore, the activation of AtHAK5 by the AtCBL9–AtCIPK1 and the AtCBL9–AtCIPK9 complexes was studied in yeast. As a control, the AtCBL9–AtCIPK23 complex, which has been also shown to activate AtHAK5 (Ragel *et al.*, 2015), was included in the study. It was observed that both AtCBL1 and AtCBL9 promoted a similar growth in the presence of 0.01 mM K^+^ of yeast cells expressing AtHAK5 and AtCIPK1 (Fig. 2). Regarding AtCIPK9, expression of AtCBL1 and AtCIPK9 together with AtHAK5 allowed yeast growth at 0.01 mM K^+^, but this did not occur when the AtCBL9 Ca^2+^ sensor was tested (Fig. 2). As shown previously (Ragel *et al.*, 2015), the two Ca^2+^ sensors also led to yeast growth at 0.01 mM K^+^ of yeast cells expressing AtCIPK23 and AtHAK5 (see below, Fig. 2).

### Transcriptional regulation of *CIPK1* and *CIPK9* in response to external K^+^

**Fig. 3.**
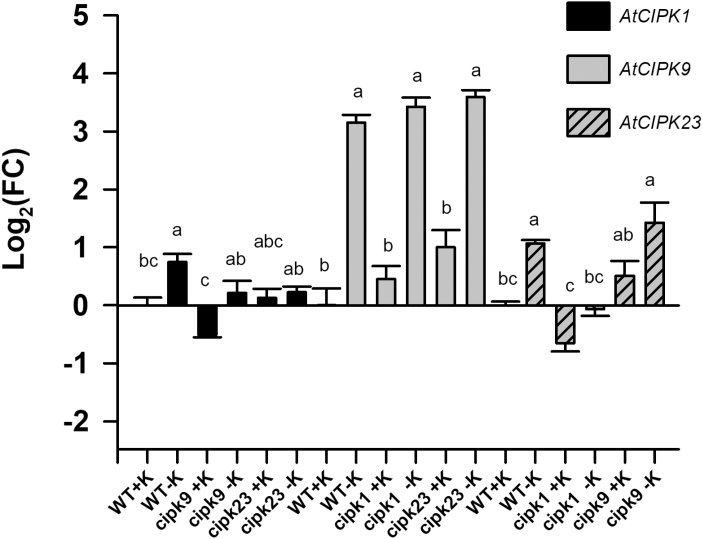
Expression levels of the *AtCIPK1*, *AtCIPK9*, and *AtCIPK23* genes in roots of WT, *atcipk1*, *atcipk9*, and *atcipk23* plants in response to K^+^ deprivation. Plants of the WT, *atcipk1*, *atcipk9*, and *atcipk23* lines were grown under K^+^-sufficient conditions for 28 d and then for 12 d in a K^+^-free solution. A set of control plants remained in the K^+^-sufficient conditions. Roots were harvested, their RNA isolated, and cDNA synthesized. qPCR was performed on cDNAs with specific primers for *AtCIPK1*, *AtCIPK9*, and *AtCIPK23*. *AtPP2A* was used as reference gene and the WT+K sample as calibrator control. Fold-change (FC) in gene expression was calculated by the 2^−^*^∆∆^*^*C*t^ method. Shown are log_2_FC average values of six independent repetitions and error bars denote standard error. Bars with different letters in data for each gene are significantly different at *P*<0.05 according to Tukey’s test.

The genes encoding the AtCIPK23 (Xu *et al.*, 2006) and the AtCIPK9 (Pandey *et al.*, 2007; Singh *et al.*, 2018) kinases were shown to be upregulated by K^+^ deficiency. The AtCIPK23 kinase was reported to activate the AtHAK5 transporter (Ragel *et al.*, 2015) and the results presented here show that AtCIPK1 as well as AtCIPK9 also activated AtHAK5 in yeast (Figs 1, 2). Therefore, the response of the genes encoding AtCIPK1, AtCIPK9, and AtCIPK23 to K^+^ deprivation was studied under our experimental conditions. Plants grown for 28 d under control conditions in 1/5 Hoagland (1.4 mM K^+^) were subjected to K^+^ starvation by transferring them for 12 d to a nutrient solution with no K^+^ added. After this time period, roots were collected, their RNA extracted, and the response of the *AtCIPK1*, *AtCIPK9*, and *AtCIPK23* genes determined by qPCR. It was observed that, in WT plants, K^+^ starvation significantly induced the expression of the genes encoding the three kinases (Fig. 3). *AtCIPK1* and *AtCIPK23* showed a mild induction (log_2_FC=0.714±0.14 and 1.06±0.06, respectively), while *AtCIPK9* showed a much higher one (log_2_FC=3.15±0.13). In addition, the expression of these three genes was also determined in the *atcipk1*, *atcipk9*, and *cipk23* mutant lines, to study possible compensatory effects derived from gene transcription. It was observed that, under K^+^ starvation, the *AtCIPK1* gene was not induced in the *atcipk9* and *atcipk23* backgrounds and the *atcipk23* gene was not induced in the *atcipk1* background. The strong induction of the *AtCIPK9* gene was not affected by knocking out the genes encoding the other two kinases.

### AtCIPK1 and AtCIPK9 activate AtHAK5 *in planta*

The results obtained in yeast suggested that AtCIPK1 and AtCIPK9 might regulate the activity of AtHAK5 *in planta*. To check this possibility Arabidopsis KO mutants with a T-DNA insertion in *AtCIPK1* and in *AtCIPK9* were characterized. For the study, Rb^+^ uptake from a 20 µM external solution was determined in K^+^-starved plants. Plants of the WT, single and double *athak5*, *akt1*, *atcipk1*, and *atcipk23* mutant lines were included as a control. The Rb^+^ uptake from a 20 µM external concentration in K^+^-starved plants, where AtHAK5 is the major contributor (Pyo *et al.*, 2010; Nieves-Cordones *et al.*, 2019), together with the KO lines employed allowed determination of whether AtHAK5 was regulated by AtCIPK1 and AtCIPK9.

**Fig. 4.**
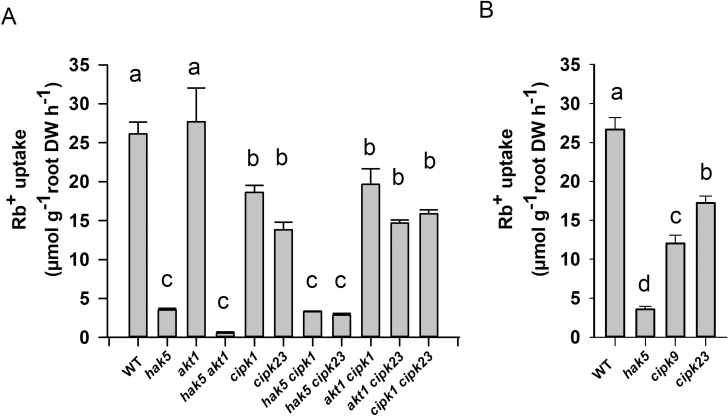
Effect of the *atcipk1* (A) or *atcipk9* (B) mutation on Rb^+^ uptake in K^+^-starved plants. (A) The effect of the *atcikp1* mutation on Rb^+^ uptake was studied by comparison with the WT, *athak5*, *akt1*, *atcipk23*, and the indicated double mutant lines. (B) The effect of the *atcipk9* on Rb^+^ uptake mutation was assayed in comparison with the control WT, *athak5* and atcipk23 lines. Plants of the indicated lines were grown for 28 d under K^+^ sufficient conditions and then for 12 d in a K^+^-free solution, and then transferred to a K^+^-free solution supplemented with 20 µM RbCl for 6 h. Plant roots and shoots were collected, dried, and acid digested to determine their internal Rb^+^ concentrations. For each repetition, the Rb^+^ accumulated in the plant was calculated by the addition of the total Rb^+^ accumulated in the root and in the shoot. The Rb^+^ uptake rates were calculated from the Rb^+^ accumulated within the plant per unit root dry weight and unit time. Shown are averages of three independent repetitions and error bars denote standard error. Bars with different letters are significantly different at *P*<0.05 according to Tukey’s test.

It was observed that, in comparison with WT plants, the absence of AtHAK5 importantly affected Rb^+^ uptake, reducing it 86.5% (Fig. 4). By contrast, the absence of the AKT1 channel did not affect the rate of Rb^+^ uptake. Rb^+^ uptake in the *athak5akt1* double mutant was almost negligible. These results showed that, under these conditions, AtHAK5 was the major contributor to Rb^+^ uptake, in agreement with previous results (Pyo *et al.*, 2010; Rubio *et al.*, 2010). The absence of AtCIPK1 or AtCIPK23 reduced Rb^+^ uptake 28.9% or 47.1%, respectively (Fig. 4A). In agreement with the idea that under these conditions AtHAK5 is the major contributor to Rb^+^ uptake, the double mutants *athak5atcipk1* and *athak5atcipk23* showed the same Rb^+^ uptake as the *athak5* single mutant while the double mutants *atcipk1akt1* and *atcipk23akt1* showed a lower rate of Rb^+^ uptake than the *akt1* single mutant (Fig. 4A). In other experiments that included the WT, *athak5*, and *atcipk23* lines as controls, the *atcipk9* line was assayed (Fig. 4B). It was observed that mutation of AtCIPK9 reduced Rb^+^ uptake 55%.

**Fig. 5.**
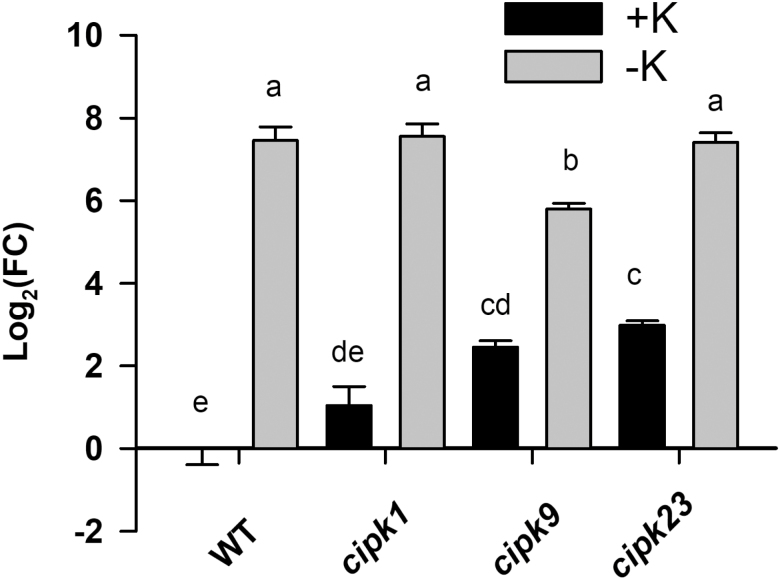
Induction of the *AtHAK5* gene in different plant lines in response to K^+^ deprivation. Plants of the indicated lines were grown and processed as indicated in Fig 3. The WT+K sample was used as calibrator control and the fold-change (FC) in gene expression was calculated by the 2^−^*^∆∆^*^Ct^ method. Shown are average log_2_FC values of six independent repetitions, and error bars denote standard error. Bars with different letters are significantly different at *P*<0.05 according to Tukey’s test.

The reduced AtHAK5-mediated Rb^+^ uptake observed in the *atcipk1*, the *atcipk9*, and the *atcipk23* lines could be due to a lower expression level of the *AtHAK5* gene in these lines. To study this possibility, the expression of *AtHAK5* was determined by qPCR in the WT, *atcipk1*, *atcipk9*, and *atcipk23* lines, under K^+^-sufficient and K^+^-starvation conditions. It could be observed that K^+^ starvation induced to the same level the expression of *AtHAK5* in WT, *atcipk1*, and *atcipk23* lines (Fig. 5). *AtHAK5* was also strongly induced in the *atcipk9* line, although to a lesser extent than in the other two mutant *cipk* lines. Interestingly, *AtHAK5* was partially induced in the *atcikp9* and *atcipk23* mutants under K^+^-sufficient conditions. In conclusion, the important reduction in Rb^+^ uptake rate shown by the *atcipk1*, *atcipk9*, and *atcipk23* mutants (Fig. 4) could not be explained by a reduced expression of the *AtHAK5* gene (Fig. 5).

### AtCIPK1 is not involved in AtHAK5 activation under hyperosmotic stress

**Fig. 6.**
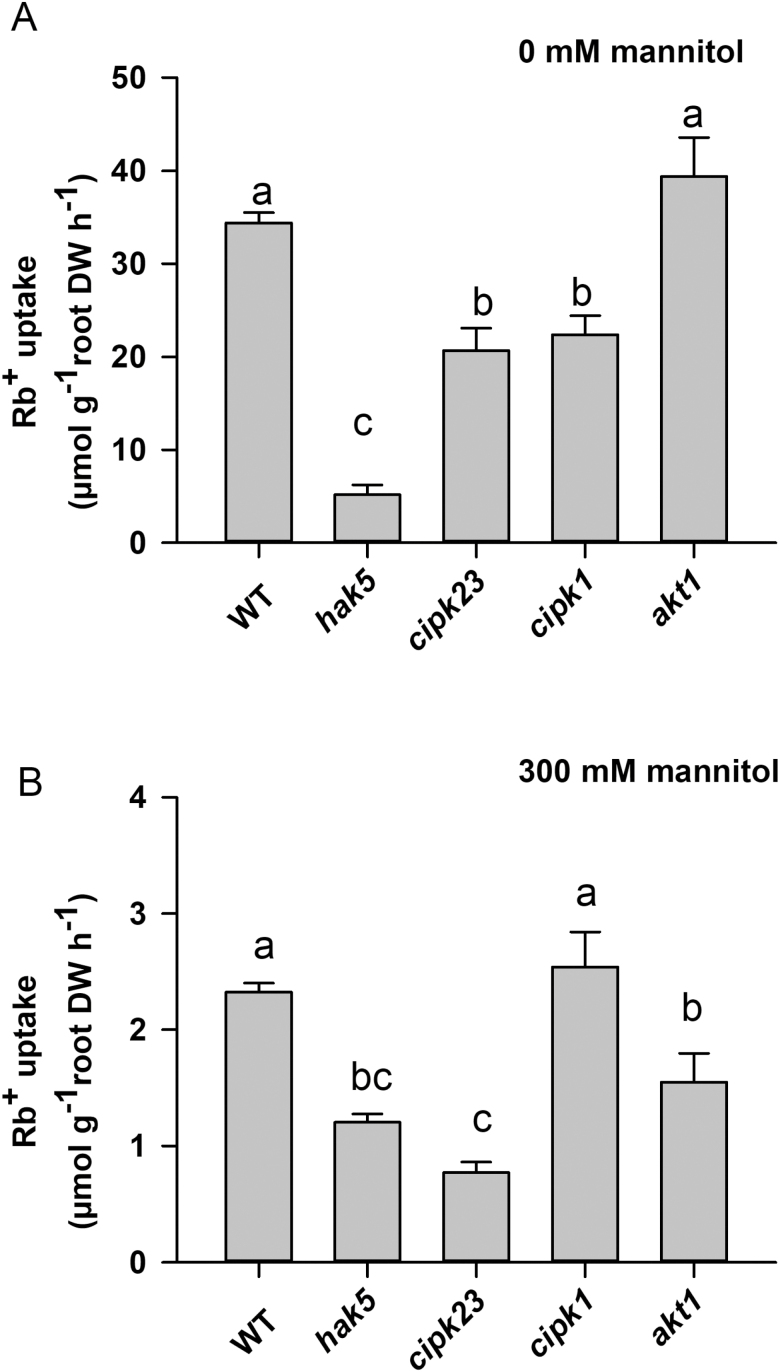
Rb^+^ uptake in K^+^-starved plants of different lines. Plants of the indicated lines were grown for 28 d in K^+^-sufficient conditions and then (A) for 12 d in a K^+^-free solution or (B) in a K^+^-free solution to which mannitol was added on days 11 and 12 of the 12 d K^+^-starvation period to reach a final concentration of 300 mM. Plants were transferred to a K^+^-free solution with 20 µM RbCl for 6 h and processed as indicated in Fig. 4 to calculate the Rb^+^ uptake rates. Shown are averages of three independent repetitions, and error bars denote standard error. Bars with different letters are significantly different at *P*<0.05 according to Tukey’s test.

AtCIPK1 has been shown to be involved in plant tolerance to hyperosmotic stress (D’Angelo *et al.*, 2006). Therefore, the possibility that this kinase regulated the AtHAK5 transporter under those stress conditions was studied. Plants of the WT and the *atcipk1* lines were grown under control 1/5 Hoagland (1.4 mM K^+^) solution conditions for 28 d and then starved of K^+^ for 12 d. In addition, a hyperosmotic stress was applied to half of the plants by adding 300 mM mannitol to the growth solution as 150 mM mannitol at each of two times, at days 11 and 12 of K^+^ starvation. After these treatments, the Rb^+^ uptake rate from a 20 µM solution was determined. As shown above, K^+^-starved WT plants showed high rates of Rb^+^ uptake, similar to those of the *akt1* plants. The *athak5* line showed very low rates of Rb^+^ uptake and the *atcipk1* and *atcipk23* lines lower Rb^+^ uptake rates than the WT line (Fig. 6). The hyperosmotic stress treatment importantly reduced the rate of Rb^+^ uptake in all plants. WT and *atcipk1* lines showed similar rates of Rb^+^ uptake, and the remaining lines showed lower Rb^+^ uptake rates than those two lines. Thus, the absence of the AtCIPK1 kinase did not lead to any effect on the accumulation of Rb^+^ when the plants were exposed to a hyperosmotic stress.

## Discussion

The high-affinity AtHAK5 K^+^ transporter is the major system mediating K^+^ uptake into Arabidopsis roots when the external K^+^ concentration is lower than 20 µM (Nieves-Cordones *et al.*, 2016; Fig. 4). Thus, its function is crucial for plant K^+^ nutrition at low K^+^ supply and AtHAK5 is activated under conditions of low external K^+^. One of the mechanisms involved in AtHAK5 regulation is its activation by the AtCIPK23 kinase (Ragel *et al.*, 2015). In addition to the AtHAK5 K^+^ transporter, the AtCIPK23 kinase has been also described to modulate the activity of several transport systems involved in the acquisition of nutrients other than K^+^ such as NO_3_^−^ (Ho *et al.*, 2009), NH_4_^+^ (Straub *et al.*, 2017) and iron (Tian *et al.*, 2016; Dubeaux *et al.*, 2018). Thus, AtCIPK23 seems to play a predominant role in regulating mineral nutrition of plants. Opposite to the mentioned activation of different transport systems by the same kinase, it has also been shown that a transport system can be activated by different CIPKs. For example, the inward rectifier K^+^ channel AKT1 is activated by the AtCIPK6, AtCIPK16, and AtCIPK23 kinases (Lee *et al.*, 2007). Thus, complex regulatory networks seem to modulate the activity of the transport systems involved in nutrient acquisition at the roots of plants.

To gain more insights into regulatory elements of AtHAK5, activation of this transporter by CIPKs other than AtCIPK23 was tested here. The 26 Arabidopsis CIPKs were screened for functional complementation in growth experiments at low K^+^ in yeast cells expressing AtHAK5 and the Ca^2+^ sensor AtCBL1. The results suggested that, in addition to AtCIPK23, other AtCIPKs could also activate AtHAK5. These kinases promoted different yeast growth capacities, from the absence of growth to vigorous growth (Fig. 1), indicating that AtHAK5 was preferentially activated by some of these kinases. A clear relationship between the growth-promoting capacity of the kinases and their phylogeny could not be observed. Thus, members belonging to the same phylogenetic group of the AtCIPK family mediated different levels of yeast complementation. These results lend support to the idea that, although phylogeny provides highly valuable information, the function of a protein cannot be directly inferred from its phylogenetic relationships and that functional tests are required for that purpose. It is worth noting that the interaction of AtHAK5 with AtCIPK23 and AtCIPK9 was not detected in previous attempts (Li *et al.*, 2006; Pandey *et al.*, 2007; Liu *et al.*, 2013) by using a yeast two-hybrid approach, for instance, to disclose physical interaction. Other approaches to study these physical interactions such as the split-ubiquitin system may be a good alternative (Grefen *et al.*, 2009). Our results indicate that the functional complementation assay for growth at low K^+^ in yeast is an appropriate method for studying AtHAK5 regulatory proteins. After the AtCIPK screening in yeast, AtCIPK1 could be identified as the kinase that produced the fastest yeast growth (Fig. 1). Importantly, AtCIPK9 has been previously reported to be involved in K^+^ homeostasis (Pandey *et al.*, 2007; Liu *et al.*, 2013; Singh *et al.*, 2018; Yadav *et al.*, 2018), and we found that it activated AtHAK5 in this initial screening (Fig. 1). Therefore, these two kinases were chosen for further characterization.

The AtCIPK screening carried out here was performed with the AtCBL1 Ca^2+^ sensor, but the two selected kinases, AtCIPK1 and AtCIPK9, have been previously reported to strongly interact not only with AtCBL1 but also with AtCBL9 (D’Angelo *et al.*, 2006; Liu *et al.*, 2013; Yadav *et al.*, 2018). Here we show that, in addition to the AtCIPK1–AtCBL1-9 complex, the AtCIPK9–AtCBL1 complex, but not the AtCIPK9–AtCBL9 complex, also led to activation of AtHAK5 (Fig. 2). The two Ca^2+^ sensors, AtCBL1 and AtCBL9, also activate AtHAK5 (Ragel *et al.*, 2015) as well as the inward-rectifier AKT1 (Xu *et al.*, 2006) via the AtCIPK23 kinase. It is worth noting that the AKT1 channel is not activated by either AtCIPK1 (Lee *et al.*, 2007) or by AtCIPK9 (Pandey *et al.*, 2007; Liu *et al.*, 2013). It seems likely that different combinations of CBLs and CIPKs compose regulatory complexes for fine tuning the modulation of root K^+^ uptake via AtHAK5 and AKT1 activities. The two Ca^2+^ sensors AtCBL1 and AtCBL9, and the three kinases, AtCIPK1, AtCIPK9, and AtCIPK23, emerge as important pieces of the regulation of K^+^ uptake at the plant roots. Since our initial screening was performed with AtCBL1, it could not be ruled out that AtCBLs different from AtCBL1 could activate AtHAK5 in combination with some of the AtCIPK kinases that did not lead to yeast complementation in our screening. This opens the modulation of AtHAK5 activity and of root K^+^ uptake to the existence of additional regulatory elements and higher levels on complexity, which deserves further investigation.

The results of AtHAK5 activation in yeast by AtCIPK1 and AtCIPK9 (Figs 1, 2) and the transcriptional induction of the *AtCIPK1* and *AtCIPK9* genes (Fig. 3) in roots by K^+^ starvation, under the same conditions that induce the *AtHAK5* gene encoding the K^+^ transporter (Fig. 5), suggested a role for these kinases in regulating AtHAK5-mediated high-affinity root K^+^ uptake. The results with the Arabidopsis KO lines (Fig. 4) confirmed such a role *in planta*. Under the experimental conditions used, AtHAK5 is the major contributor to Rb^+^ uptake (Fig. 4; Pyo *et al.*, 2010; Nieves-Cordones *et al.*, 2019). The *atcipk1* and *atcipk9* mutations reduced the rate of high-affinity Rb^+^ uptake 28.9% and 55% with respect to WT, respectively (Fig. 4). Mutation of the *AtCIPK1*, *AtCIPK9*, or *AtCIPK23* gene had a small effect on the up-regulation of the *AtHAK5* gene induced by K^+^ starvation (Fig. 5). Similarly, mutation of *atcipk1*, *atcipk9*, or *atcipk23* had a small effect on the expression of the genes encoding the other two kinases (Fig. 3). These small effects of mutating one gene on the expression of the other genes (Figs 3, 5) could not explain the important reduction in the Rb^+^ uptake when one AtCIPK was mutated (Fig. 4). Thus, the most likely explanation for our observations is that, when AtCIPK1 or AtCIPK9 is lacking, the activity of the AtHAK5 transporter is reduced and it can be concluded that AtCIPK1 and AtCIPK9 regulate AtHAK5-mediated high-affinity K^+^ uptake at the root.

Previous studies have shown that AtCIPK1 was required for osmotic stress tolerance (D’Angelo *et al.*, 2006). By forming alternative complexes with AtCBL1 or AtCBL9, the kinase could regulate ABA-independent and -dependent responses to abiotic stress. These complexes were shown to be located at the plasma membrane where, as suggested by the authors, they would regulate a plasma membrane ionic transport system. However, no final target for AtCIPK1 kinase was identified in that study. Other studies have related AtCIPK9 function to the maintenance of K^+^ homeostasis (Pandey *et al.*, 2007; Liu *et al.*, 2013; Singh *et al.*, 2018). Similarly to the studies on AtCIPK1, the studies on AtCIPK9 propose that this kinase may regulate a K^+^ transport system, but no final target was identified. Here we identify a target of AtCIPK1 and AtCIPK9 and present physiological and genetic evidences that these two kinases regulate the activity of the AtHAK5 K^+^ transporter *in planta*. Regulation of AtHAK5 by AtCIPK1 seems to be unrelated to the role of this kinase in osmotic stress tolerance because we could not observe an effect of AtCIPK1 in AtHAK5-mediated K^+^ uptake under hyperosmotic stress conditions (Fig. 6). Interestingly, loss of AtCIPK23 reduced K^+^ uptake in both the absence and the presence of osmotic stress (Fig. 6) suggesting that this kinase regulates AtHAK5 under a wider range of conditions than AtCIPK1, something that deserves further investigation.

The results presented here show different levels of yeast complementation by AtCIPK1, AtCIPK9, and AtCIPK23 (Fig. 1). From these differential effects, it is tempting to assign these kinases different levels of contribution to AtHAK5 regulation. However, it should be taken into account that important compensatory effects may take place when one of these kinases is knocked-out in the plant and the relative contribution of each of them to AtHAK5 regulation is difficult to establish. The picture that emerges from many other studies and the present one is that different CBL Ca^2+^ sensors may combine with different CIPK kinases to form complexes that regulate specific protein targets. This plethora of arrangements may provide the molecular mechanisms for the fine regulation of their target proteins.

## Supplementary data

Supplementary data are available at *JXB* online.

Table S1. Primers used for determination of expression levels of *AtHAK5*, *AtCIPK1*, *AtCIPK9*, and *AtCIPK23* by qPCR.

eraa212_suppl_Supplementary_Files
