# Supplementary material for: Arabidopsis K+ transporter HAK5-mediated high-affinity root K+ uptake is regulated by protein kinases CIPK1 and CIPK9
Source: J Exp Bot. 2020 Jun 2;71(16):5053–60. doi: 10.1093/jxb/eraa212 (PMC7410179; doi:10.1093/jxb/eraa212)
Supplement: eraa212_suppl_Supplementary_Files [file eraa212_suppl_supplementary_files.pdf]

Supplementary Table 1. Primers used for determination of expression levels of *AtHAK5*, *AtCIPK1*, *AtCIPK9* and *AtCIPK23* by qPCR

Gene	Primer name	sequence
AtPP2A	AtPP2AF	5´-TAACGTGGCCAAAATGATGC-3´
AtPP2A	AtPP2AR	5´-GTTCTCCACAACCGCTTGGT-3´
AtHAK5	AtHAK5F	5`-AAGAGGAACCAAATGCTGAGACA-3´
AtHAK5	AtHAK5R	5´-GCCCCGATGAAGGGACAT-3´
AtCIPK1	AtCIPK1F	5´-CGTCACTGAACGTGGTTGAG-3´
AtCIPK1	AtCIPK1R	5´-TACTATCTCTTGCTCCGGCG-3´
AtCIPK9	AtCIPK9F	5´-GAGTTTTAGCTGCCCACCATG-3´
AtCIPK9	AtCIPK9R	5´-GGTTGGGTTCGAGAATACGC-3´
AtCIPK23	AtCIPK23F	5´-CGACATCCCAGGGTCTCAA-3´
AtCIPK23	AtCIPK23R	5´-GTTTCACCAGCCCCATTTGT-3´
